# Harley's Course—Integrating Teachings From Western and Indigenous Sciences in an Undergraduate Biology Course

**DOI:** 10.1002/ece3.71824

**Published:** 2025-07-16

**Authors:** Carol L. Armstrong, Alexandria Farmer, Michelle Hogue, Harley Bastien

**Affiliations:** ^1^ Department of Biology Mt Royal University Calgary Alberta Canada; ^2^ Department of Indigenous Studies University of Lethbridge Lethbridge Alberta Canada; ^3^ Piikani Knowledge Holder, Blackfoot Confederacy Brocket Alberta Canada

**Keywords:** experiential, land‐based learning, perception, relationship, resistance

## Abstract

What is science? Whose knowledge do you value and why? Is there room for spirituality in science? These are core questions in the third‐year biology course officially titled Common Ground: Learning from the Land (BIOL3201) offered at Mt. Royal University (Calgary, Alberta, Canada). Commonly referred to as “Harley's Course”, this course was co‐developed with Piikani Knowledge Holder Harley Bastien. The purpose of the course is to expose students to comparative scientific perspectives—Indigenous perspectives based on relationships with creation and respect for the natural order of life, with western perspectives based on maximizing land productivity and management. It encourages students to challenge their beliefs about what science is, who is a scientist, what it means to “think scientifically”, how to listen and observe, and the validity of the immeasurable. The opportunity to experience relational land‐based learning, and to have the flexibility and freedom to discuss and reflect on perspectives different from the dominant western perspective has a remarkable impact on the students. This paper includes lessons learned from the first three cohorts of students who participated in “Harley's Course” and shares some of the challenges inherent in decolonizing the western post‐secondary science curriculum.

## Graphical abstract

This paper describes a relational land‐based learning experiential field course that bridges Western and Indigenous scientific worldviews. We describe the impact of incorporating traditional Indigenous paradigms and knowledge into post‐secondary science curriculum and the challenges inherent in decolonizing Western science.

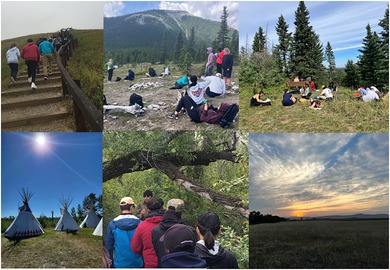

## 1 Introduction

There are plenty of spirits in the valley of Napii eek stunn [Oldman River] that flows east from the Rocky Mountains through southern Alberta. The tipis stand tall, grouped together on the west side of camp, surrounded by appin niigii [wild prickly rose, *Rosa acicularis* ], buckgiibii [choke cherry, *Prunus virginiana* ] and otsiibii [willow bushes, *Elaeagnus commutata* ]. To their left, tucked into a grove of oppagii [balsam poplar, *Populus balsamifera* ] is the cook tent and picnic tables and a firepit that crackles and smokes as the wet wood burns. Stookatsis [purple clematis, *Clematis occidentalis* ] creeps up the oppagii [poplar trees, *Populus balsamifera* ] at densities not observed anywhere else, and its huge clumps of Ghost Whisperer seeds wait for the next gust of wind to blow them away. We listen to the river. Today it sings loudly as the rain pours from Grandfather sky, and the water swells as it hurries towards the east direction. The dark clouds are so close we can reach for them. *Souta gii* [Rainwoman] is here.

How does this story begin? In 2018, a small group of faculty members from the Faculty of Science and Technology at Mount Royal University in Calgary, Alberta (Canada) spent a night at Harley Bastien's Buffalo Rock Tipi camp along the shores of Napii eek stunn [Oldman River]. Harley, a Piikani Knowledge Holder from the Blackfoot Confederacy, was our guide, our storyteller, our host. Harley is part of Napii eek stunn [Oldman River valley]. The DNA of his ancestors is part of the old cottonwood trees that line the river bank and their mystical spirits surround us. This experience was the beginning of what would become a third‐year undergraduate biology course called *Common Ground: Learning from the Land* (BIOL 3201) – or colloquially and in respect and gratitude for Elder Harley, simply “Harley's Course”.

### 1.1 Course Creation

“Harley's Course” was created in response to the Truth and Reconciliation Commission of Canada's Calls to Action to educate and build student capacity for intercultural understanding, empathy, and mutual respect (#63.C, 2015). In 2018, the Office of Academic Indigenization (OAI) co‐led by Drs. Renae Watchman and Liam Haggarty, hosted an Indigenous Curriculum Development Gathering that provided faculty and Indigenous communities the opportunity to work together to build the framework for the Indigenization of Mount Royal University's curricula. Faculty members and Community Partners were paired with each other and tasked with developing a full 13‐week course outline and syllabus that includes Indigenous knowledge and pedagogies while still meeting course requirements. Decolonizing the dominant Euro‐western curriculum is challenging at best and particularly in the sciences at the post‐secondary level. We are grateful for the combined efforts of the OAI, Charles Hepler, Khatija Westbrook, Dorothy Hill, and Melanie Rathburn at Mount Royal University in the initial stages of course development. We are especially grateful to Piikani Knowledge Holder Harley Bastien for his leadership, engagement, and love of the land that led to the creation of this course.

### 1.2 Course Development

**FIGURE 1:**
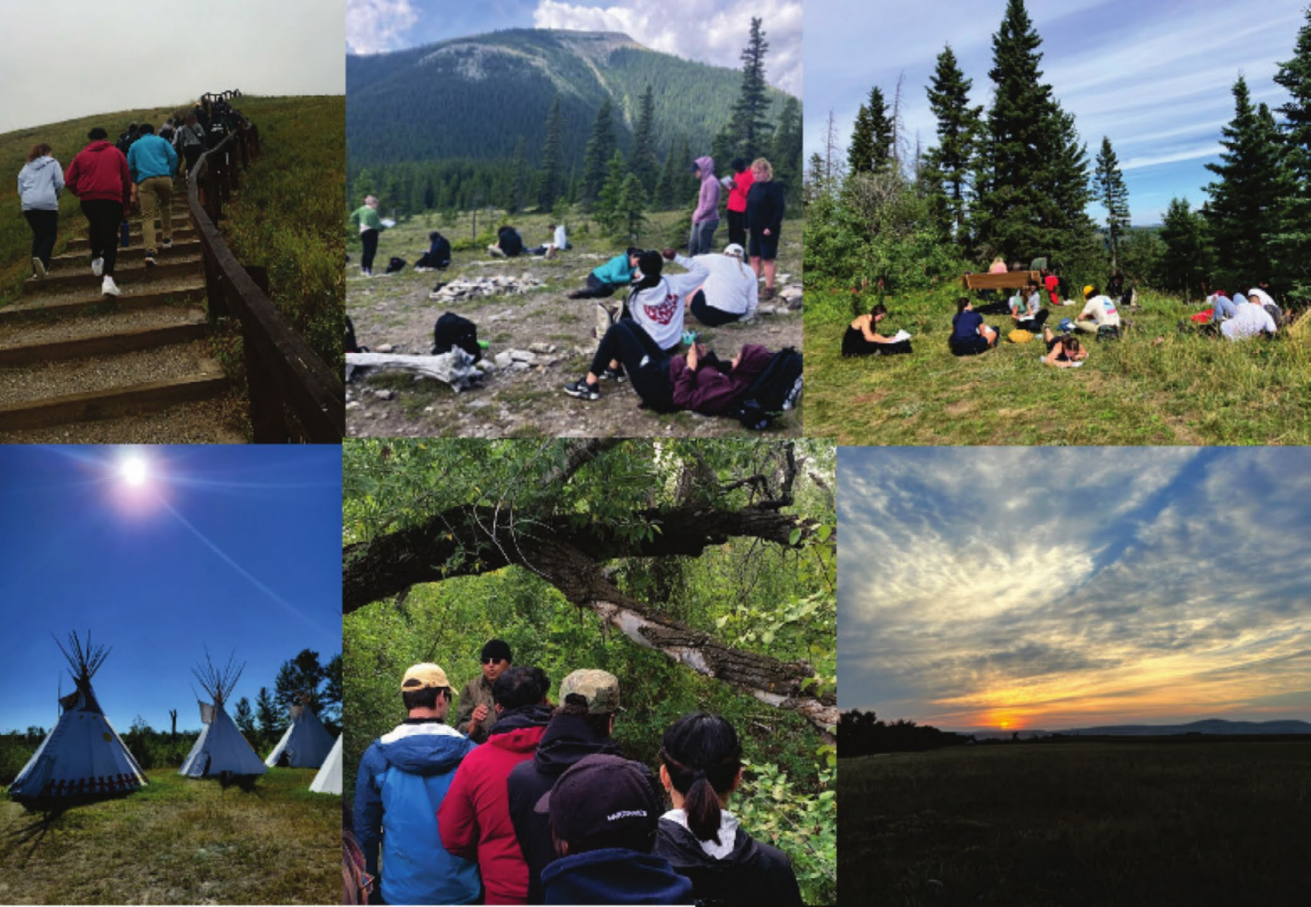
Graphical abstract of “Harley's Course”.

The development and structure of “Harley's Course” was, and is, fluid and each offering of the course has been slightly different. The students taking this course are typically in their fourth or fifth year of undergraduate studies and have prior knowledge in ecology from a second‐year pre‐requisite course titled Principles of Ecology and Evolution (BIOL 2213). They have a strong foundation in western ecology, having studied the scientific method, the main concepts of ecology at the individual, population, community, and ecosystem levels, and learned to collect, organize, manipulate, and analyze ecological data. In the academic calendar, the course is labeled as a third‐year Biology course and described as follows: this course provides an introduction to the common ground between western and Indigenous scientists with a focus on learning from the land. Students will explore traditional territory of the Piikani people adjacent to the Napii eek stunn [Oldman River] with both an Indigenous scientist and a western‐trained scientist in order to gain perspective on what science is, how to listen and observe, the validity of the immeasurable and scientific sources. The course learning outcomes state that on successful completion of the course, students will be able to provide a working definition of a scientist and science worldview, recognize the unique entities of both western and Indigenous science and thoughtfully explore the common ground between them, demonstrate a respectful understanding of diverse perspectives in order to increase scientific literacy, and develop a holistic view of science through a better understanding of the interconnectedness of the natural world. “Harley's Course” is offered as an immersive 3‐week block course prior to the start of the Fall semester: the first 2 weeks are in person and comprised of daily field trips to nearby parks, farms, mountains and Blackfoot Heritage sites as well as the opportunity to spend 3 days and two nights camping at Harley's Buffalo Rock Tipi Camp on the Piikani Reserve on Treaty 7 territory in Southern Alberta, Canada, while the third week is for students to independently complete their final projects (Figure 1).

### 1.3 Education and Ways of Coming to Know

There are as many ways to learn about the common ground between western and Indigenous science as there are coffee grounds in a pot of shared coffee and we acknowledge that there are many academics investing positive energy into responding to the 2015 Truth and Reconciliation Commission of Canada Calls to Action through innovative, collaborative and experiential land‐based learning (e.g., Michie et al. 2018; Sanderson et al. 2020). When it comes to teaching and learning about Indigenous science, we have listened to and honored the stance of “nothing about us without us” (Bridges and Bridges 2017) by connecting and working with Blackfoot Knowledge Holders, a Blackfoot historian at the site of the signing of Treaty 7 (Blackfoot Crossing Historical Site, Siksika, Alberta) and Blackfoot Elders at the UNESCO World Heritage site Head Smashed‐In Buffalo Jump (west of Fort MacLeod, Alberta, Canada where “the foothills of the Rocky Mountains begin to rise from the prairie” (Wikipedia 2025)). We have also been welcomed by, and learned from, buffalo farmers, cattle ranchers, and cooks and hold space for and respect their experiential knowledge and expertise.

The purpose of this paper is to share some of the lessons learned during the first 3 years of “Harley's Course” (2022–2024) and our approach to developing a course that bridges Indigenous and western scientific knowledge on the land. As non‐Indigenous educators, we wanted to build an experiential field course that encouraged connection with the natural world with heart and mind and spirit and body, recognition as a contrast to the colonial framework that dominates post‐secondary science curriculum and engagement of students as participants rather than passive observers. We were guided by the C4‐R4 methodology for meaningful and authentic collaboration between Indigenous and non‐Indigenous scientists that emphasizes Co‐learning Co‐designing Co‐creating and Co‐sharing along with the 4 R's (Respectful relationships, cultural Relevance, Responsibility and Reciprocity) (Hogue 2025, personal communication). The Indigenous philosophies of relationship and reciprocity were key in the development and implementation of this course (Table 1).

**TABLE 1:** Course overview. BIOL3201 common ground: learning from the land. “Harley's course”.

*Building relationship*		
Pen and paper in‐class writing assignments based on Kimmerer's *Braiding Sweetgrass* text	Self‐localization essay to situate yourself	Cooperative land‐based scavenger hunts
*Learning from the land*		
Land acknowledgements (Whitmore and Carlson 2022)	Hike to the Siksikaitsitapi medicine wheel at Ootssapi'tomowa (Nose Hill, Calgary)	Buffalo as Teachers: field trip to a buffalo farm and UNESCO site Head Smashed‐In Buffalo Jump
Land as our Textbook (hike in Kananaskis Country)	Plants as Our Relatives (Cooper et al. 2025)	Buffalo Rock tipi camp overnight trip with Piikani Knowledge Holder Harley Bastien (3 days)
*Spirituality in science*		
Etuaptmumk (two‐eyed seeing) Bartlett et al. (2012)	Talking circle	Transformative change
*Reciprocity*		

### 1.4 Relationship

Our endeavor to design and implement an authentic, reconciliatory, transformative land‐based learning course started with our connection and relationship with Harley Bastien and his experience as an Indigenous scientist and Piikani Knowledge Holder. As academics trained and accustomed to post‐secondary institutions and timelines, we needed to replace the pervasive “get work done” attitude with understanding, patience, and an acknowledgment that there is much to learn in order to do things in a good way (Zeidler 2011). As Battiste (2019) states, “part of the ultimate struggle is a regeneration of new relationships among and between knowledge systems, which needs scholars competent in both knowledge systems to converge and reconcile these and other knowledges, ways of knowing, and systems” (103). This means setting aside the dominant lens of curriculum development, letting go of ego, embracing humility and listening. In the Indigenous paradigm, a commitment to relationship also means listening and paying attention to the river and the valley, the mountains and the rocks, the plants and the standing ones (trees) and respecting their contributions. Relationship is about stepping into the “vulnerability of shared space” (Snow 2023).

Relationships are not a key part of western science. In fact, the core concept of neutrality and objectivity discourages the development of relationships and as a result, an objective passivity has been instilled in western‐trained science students (Aikenhead 2001) that manifests as resistance to considering alternate epistemological perspectives. The challenge of engaging students in a relational way is apparent from the first day of the course as many of the students attend the same university in the same degree program, yet are reluctant to interact with one another. We spend the first part of the day building relationships with each other and the land through a variety of activities such as scavenger hunts and traditional stories. We introduce the Indigenous sacred medicines and offer tobacco to the earth in reciprocity and build a relational twine spiderweb, that gives each student the opportunity to introduce themselves, share how they are feeling about joining the course and connect with others. We find that the students are very honest and up front about their decision to take the course, with many of them admit to wanting to “hang out outside” or “get an easy A” (Table 2). This misconception and misunderstanding about the course requirements are slowly shifting as more students complete the course and discuss it among themselves and appreciate the unique aspects of this course.

**TABLE 2:** Students' reasons for taking BIOL3201 as shared in their self‐localization essays.

2022	2023	2024
To be completely honest, I originally enrolled in this course because I was looking for a block week course so that I could graduate after Fall semester	I would be lying if I said that the idea of a block week course lightening the load over the rest of the term didn't sweeten the pot	This course could be used as one of the approved options that align with my program's requirements
I took this course so that I don't have to take 5 courses next semester	To learn firsthand to appreciate and form relationships with the land	It would be easy and I love being surrounded by the outdoors
I took this class because I need to finish my degree	Strengthen my connection with the land and go beyond taxonomic classification	I like to learn on the go. I much prefer to learn on my feet rather than sitting in the classroom and reading textbooks
I took this course so I could graduate in the Spring	Gain a deeper understanding and appreciation for Indigenous perspectives on science, Indigenous knowledge, and Indigenous belief systems	This course is mandatory for my program and it pushes me out of my comfort zone
This course will look good on my med school application	Find connections between my life and the narratives of others in the world	To learn from doing things hands‐on and see it in real life rather than in a textbook
Learn from a genuine indigenous teacher about the values, beliefs, and lifestyle of the First Nation people	Understand my role in the ecosystem	Gain a new perspective and appreciation for non‐Western approaches to science and medicine
I wanted to develop a deeper understanding of indigenous teachings	I wanted to enrich my understanding of the world and to shape my capacity for empathy and critical thinking	I enrolled in this course with the intention of expanding my knowledge of the individuals whose histories are interconnected with the place I currently call home
To learn more about Indigenous ways of knowing and different perspectives on science	I want to broaden my understanding	
Wanting to spend more time in nature	Living next to the TsuuT'ina Nation I realized I knew nothing about them	

### 1.5 In Class Writing Assignment/Reflection and Challenges

One of the first steps in shifting perspective in “Harley's Course” involves finding a quiet place to think and putting pen to paper. Since the entire course is held outdoors, that means that the in‐class writing assignments are also done outside—sometimes in the rain or cold or heat depending on the weather. Students are assigned chapters from *Braiding Sweetgrass: Indigenous Wisdom, Scientific Knowledge and the Teaching of Plants* (Kimmerer 2013) and then given a set of prompts/questions to write about (see Table 3). The students are accustomed to using computers and tablets to write assignments and it is a real adjustment for them to use pen and paper and not be able to edit their work as they type. During the in‐class writing assignments, it was very common for students to express concern that they were contradicting themselves within an answer—their minds are changing literally as they are writing—and they are skeptical when told that is a powerful and positive thing! It is also an adjustment for science students to write reflectively and personally, rather than from a third‐person removed lens that is considered “scientific writing”, and to respond to open‐ended questions that have no “right answer”. One of the biggest challenges with the in‐class writing assignments was encouraging students to answer difficult questions that required self‐reflection in a genuine manner rather than providing the answer they think we want to hear. For example, on the first day of class, after reading Kimmerer's well‐known passage describing both her desire to study botany “to learn about why asters and goldenrods look so beautiful together” (Kimmerer 2013, 39) and the subsequent response from her academic advisor who told her in no uncertain terms that was “not science” (2013, 40), the students are asked to answer the questions: “What is science? Are you a scientist?”. These simple questions are deceptively hard to answer and inevitably stump the students who are in their final years of undergraduate studies and have completed more than 20 science courses. As expected, all of the students defined science as rational, logical, methodical; a rigid approach to test hypotheses and analyze data. Surprisingly, more than 70% of the students said they were not scientists (Table 4).

**TABLE 3:** A few examples of prompts/questions based on assigned readings (chapters from Braiding Sweetgrass: Indigenous Wisdom, Scientific Knowledge and the Teaching of Plants, Kimmerer 2013).

Asters and goldenrod	“Why two flowers are beautiful together would violate the division necessary for objectivity” (42). Is objectivity and neutrality necessary in science? Why or why not?
Council of Pecans	Do you agree or disagree with the statement that “science pretends to be purely rational, completely neutral, a system of knowledge‐making in which the observation is independent of the observer” (19)
Learning the Grammar of Intimacy	The author notes “science can be a language of distance which reduces a being to its working parts; it is a language of objects.” … “in scientific language our terminology is used to define the boundaries of our knowing” (49). Do you agree with these statements?
Epiphany of Beans	In this chapter, during a writing workshop on ‘relationships to the land’, the students expressed a love of nature. The students were then asked “do you think the earth loves you back?” (124). What is a love of nature? Can a planet love you? Discuss this prompt with respect to both western and Indigenous perspectives

**TABLE 4:** Science student responses to the prompt given on first in‐class writing assignment: Are you a scientist?

No‐because…	Yes‐because…
I am only a pseudoscientist	I am inquisitive
I need logical reasoning	I question the world
I need time in the field	
I don't have a degree	
I need to do research	
I am still in training	
I haven't discovered anything new or unexplained	
I am only a mini scientist	
I haven't published	
I haven't written a peer‐review article	

The strict definitions of what constitutes scientific inquiry, what the “five characteristics of science” are (reproducible, testable, tentative, predictive, and explanatory) and who is considered elite or accomplished enough to call themselves a scientist is deeply instilled in colonial‐focused education systems (Snively and Corsiglia 2016, 1). Starting here helps us encourage the students to consider why these constructs are in place, who put them there and if they make sense. In addition to the rigid beliefs of what science is, and what it is not, students are also trained in black and white thinking—something is right or wrong and arguments require evidence. It is hard to “reconceive what we thought we knew” (Battiste 2019, 124) and shift ingrained views about science. As one student commented in their reflections,

I will admit that I viewed science as either being done correctly or incorrectly, with little wiggle room in between. (Harley's Course, 2022)

### 1.6 Self‐Localization Essay

In the Indigenous paradigm locating oneself is important as it provides a context of who one is in relation to the context they are currently in. To do this, Indigenous peoples will often introduce themself in relation to their family and their community and then talk about themself. In this context, after their first day in the course, the students write a 4‐page self‐localization essay in response to, and guided by, a prompt taken from Robin Wall Kimmerer's text Braiding Sweetgrass: Indigenous Wisdom, Scientific Knowledge and the teachings of Plants (2013). In this prompt, Kimmerer explains that “the Skywoman story, shared by the original peoples throughout the Great Lakes, is a constant star in the constellation of teachings we call the Original Instructions. These are not “instructions” like commandments, though, or rules; rather they are like a compass: they provide an orientation but not a map. The work of living is creating that map for yourself” (Kimmerer 2013, 7). In the self‐localization assignment, students are asked to write about exploring their “map”: who they are, their past and their present, their language and values, where they live, where they are from and why they are taking this course. The assignment also asks the students to describe their knowledge and understanding of where they live (Treaty 7 territory), their role as treaty people and Indigenous ways of knowing. Treaty 7 was an agreement signed on September 22, 1877, between the Canadian Crown and several First Nations in Southern Alberta, including the Siksika, Kainai, and Piikani (Blackfoot Confederacy), the Îyârhe‐Nakoda and the TsuuT'ina people, to share the land and resources between the Rocky Mountains (west) to the Cypress Hills (east), and from the Red Deer river (north) to the United States of America border (south). As the Elders say: *start with where you are*. The treaties are in place as long as the sun chines, the grass grows and the rivers flow, yet many Canadians are unaware of their treaty responsibilities due to the impacts of colonization. The self‐localization essay is an opportunity for self‐reflection and awareness which also prime western‐trained university students to have a broader understanding of their emotions and reactions to dissident ways of knowing. The students enjoy this assignment and it is an important foundation for the relational work in the course.

## 2 Course Activities: Learning From the Land

All ten days of Common Ground take place are outdoors: challenging contemporary notions of classrooms, we intentionally acknowledge and recognize the land as our teacher (Cooper et al. 2025). By spending time outside and participating in land‐based activities such as nature walks, mountain hikes and off‐grid tipi camping, the students learn to pay attention to, and adjust their pace to synchronize with, the world around them. Learning from the land means taking the time to recognize the plant relatives and animals and elements, and acknowledging that they have much to teach us. Learning from the land also includes listening to difficult lessons that may come from the miiyíkssopoyi [strong wind] as it pulls up your tipi pegs in the middle of the night, from sootaa [rain] as torrential sheets of water pour from the sky to drench your camping gear, from sstsi'kitsi [searing heat], from áípapommiksi [lightning flashes] that fill the horizon, and from the rushing current of niítahtaa [river] as it carries you away. These lessons are as important as those learned from texts and instructors in a traditional ecology classroom. While western science is often a commensal relationship, whereby one species (humans) typically benefits while the other species (nature in general) is either unaffected, impacted, or harmed, land‐based learning represents a far more mutualistic relationship in that humans are part of an interconnected whole that recognizes the spirit and life within everything. The following Learning from the Land course activities were designed to promote mutualism and connection with the land.

### 2.1 Land Acknowledgements

Land acknowledgements are an intentional acknowledgment of the historical and current stewardship of the land by the First Nations people. They recognize our relationship to the land and to one another, help situate us “where we are” (Whitmore and Carlson 2022) and serve as a “stepping stone to honouring broken treaties” (Mills 2019). Each day of the course, the first step in connecting to the land was to offer a land acknowledgment. All of the students have heard land acknowledgements before—at hockey games, concerts and public events—but like many, they dismiss it as a performative and somewhat meaningless statement. When we took the time to talk about the treaties, the original stewards of the land long before non‐Indigenous people were here, and the fact that land acknowledgments are “an attempt to draw historical relationships into intercultural dialogue in order to decolonize assumptions about ownership and place and explain that they are entry points into relationship with Indigenous neighbours and meant to facilitate engagement” (Snow 2023), the students had a better understanding of the why they were important but were still incredibly fearful about the how to do them. As one student said,

When asked to do a land acknowledgement I felt profoundly lost, unsure of who or what I was supposed to acknowledge, terrified of getting it wrong and disconnected from the land around me. I am a longtime hiker but this was a fundamental shift in how I saw the environment. (Harley's Course, 2023)

To address this fear, which is also present in many of my science colleagues, we taught them the proper protocol of offering tobacco as a sacred medicine to honor the land with an acknowledgement or prayer, and encouraged them to situate themselves within their physical surroundings. For example, as a group, we created acknowledgments according to our location—the riparian zone beside the Bow River at Blackfoot Crossing (Siksika Nation), on a grassy hillside above the Elbow River adjacent to the Tsuut'ina Nation, in the Rocky Mountains of Kananaskis Country (Îyârhe Nakoda Nation), in a manicured city park, or in a prairie field filled with stone tipi rings (Piikani Nation). Connecting ourselves to the land meant that land acknowledgements became meaningful and authentic instead of ritualistic, rote or scary, and many of the students commented on this in their reflective journals.

### 2.2 Hike to the Siksikaitsitapi Medicine Wheel

Early in the morning on the second day of class, we hike through the native grasses and rough fescue grassland to the Siksikaitsitapi Medicine Wheel at Ootssapi'tomowa (Look Out Hill/Nose Hill, Calgary, AB), a ceremonially significant and sacred site of reflection (Pard et al. 2016). As we sit beside the ring of sacred stones, the Rocky Mountains to the west and the Bow River Valley and plains to the east, we begin to strengthen the fragile web of relationship to each other and to the land that was created the day before and ask each student to introduce another student in the course. We also spend time in silence, listening to the leaves of trembling aspen, (Populus tremuloides, Blackfoot oppagii) that line the coulees and shelter the white‐tailed deer ( *Odocoileus virginianus* , Blackfoot áwatoyi), mule deer ( *Odocoileus hemionus* , Blackfoot isspaysstoo), coyotes ( *Canis latrans* , Blackfoot aapí'si), porcupines ( *Erethizon dorsatum* , Blackfoot kaaysskááhpiksi), northern pocket gophers ( *Thomomys talpoides* , Blackfoot nato's) and Richardson's ground squirrels ( *Urocitellus richardsonii* , Blackfoot omahkokata). Above us soar Northern Harriers (*Circus hudsonius*, Blackfoot aapiipissiyoohsi) and Swainson's hawks ( *Buteo swainsoni* , Blackfoot sikohtapikaiimiiksi) (City of Calgary 2025). As a class, we spend time here reconnecting, acknowledging the land, and learning about medicine wheels (Pard et al. 2016; Mashford‐Pringle and Shawanda 2023; City of Calgary 2025). We discuss the four quadrants within the circle of medicine wheel and ask if the Indigenous approach in which we “understand a thing only when we understand it with all four aspects of our being: mind, body, emotion and spirit” (Cajete 1994) can be applied to science, or if, as Kimmerer says, “science privileges…only mind and body” (Kimmerer 2013, 45). During our class session at the Siksikaitsitapi Medicine Wheel, we ask the students to consider a scientific problem using mind and body and emotion and spirit, which often leads to discomfort: as one student said.

I was uncomfortable at the medicine wheel. I was worried about messing up and uncomfortable thinking about my problem from all 4 aspects. To think so critically about my life really affected me and I drove home that day crying tears of relief and hope. (Harley's Course, 2023)

The opportunity to use “all 4 aspects” creates an opening for the students who have been repeatedly told that there is no place for emotion or spirit in science.

### 2.3 Buffalo as Teachers

The importance of the iinii [buffalo, *Bison bison* ] to the Plains Indigenous people is highlighted at Blackfoot Crossing Historical Park (Siksika, Alberta) and the UNESCO World Heritage site Head‐Smashed‐In Buffalo Jump (near Fort MacLeod, Alberta). In anticipation of our excursions to these sites later in the course, we arranged for the students to tour a farm in order to experience a western approach to working with buffalo. After touring the pasture and spending time with these majestic creatures, the students explore the beautiful cascading mutualism of buffalo with their ecosystem: the wallows that become temporary water holes that change the microclimate and conserve water (Frasier 2025), the dung and urine that are important sources of nitrogen, phosphorus, calcium, sulfur, and magnesium for microbes, plants, butterflies, birds, and other animals (National Park Service 2016) and the clever role of the dung beetles that methodically bury the dung to return these nutrients to the land (National Park Service 2016). Buffalo feces support ants that in turn are eaten by Northern Flickers ( *Colaptes auratus* , Blackfoot pii'ka) (Olsen 2022) and the patties are used by Burrowing Owls ( *Athene cunicularia* , Blackfoot maatáásiiksi) to line their nests (Grueskin 2023); when buffalo hair is used to line nests of tallgrass prairie birds, it increases nesting success (Coppedge 2009). This trip gave students the opportunity to integrate their textbook knowledge about ecological relationships with the complex and nuanced relationships experienced in the field.

### 2.4 Hike in Kananaskis Country

**FIGURE 2:**
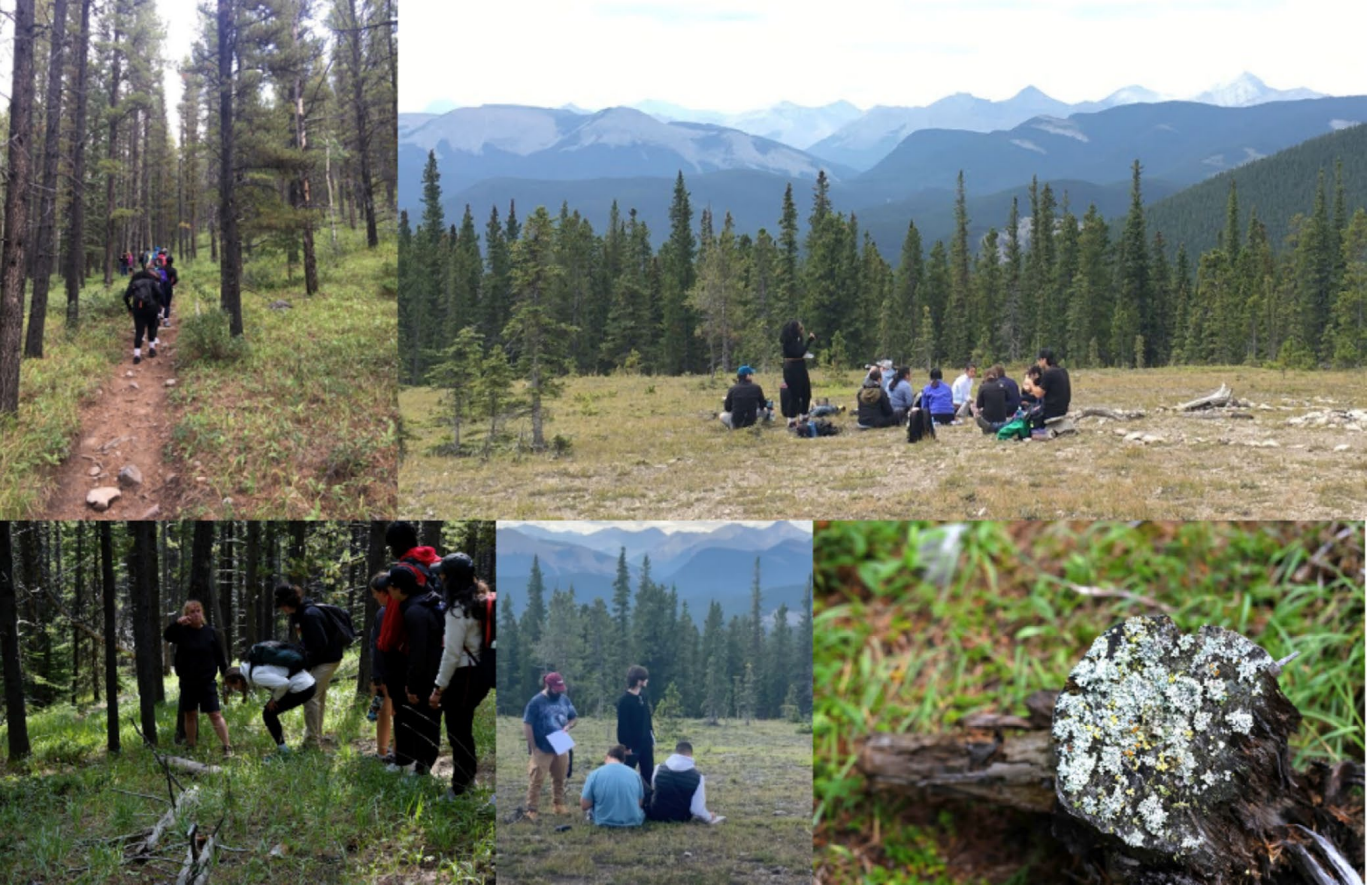
‘Land as our Textbook’ Hike in Kananaskis Country.

A “Land as Our Textbook” relational learning activity was centered around a ten‐kilometer guided hike in the foothills and mountains of Kananaskis Country west of Calgary with a western‐trained ecologist (Figure 2). In preparation for the hike, the students were asked to read the chapter *Witness to the Rain*, (293–300) (Kimmerer 2013) that illustrates how observations and curiosity can lead to scientific questions. In *Witness to the Rain*, the author notices (while lying under a fallen log in the woods) that “the droplets [of water] on *Isothecium* are far bigger than the drops on my bangs” (294) which leads to the hypothesis: “maybe [while] in residence in mosses, raindrops absorb some property that increases their surface tension, making them stronger against the pull of gravity” (294). Similarly, during the hike up the mountainside, the students were asked to tap into their somatic senses and notice/be mindful of at least three observations (flower colors, tree heights, soil texture, lichen patterns, animal footprints, air temperature etc.). Then, at the midway point of the hike, sitting in circle in a meadow surrounded by stunted spruce and looking out over the tree‐covered hillside of Nihani Ridge and Forget‐me‐Not Mountain, the students were asked to share some of these observations or questions with the class. Rather than offering genuine expressions of curiosity and wonder at the landscape they had just hiked over, we found that students would instead simply echo the questions that had been offered by the ecologist that guided them up the mountain, suggesting that the ability to be open and simply observe with an intuitive investigative spirit is dampened through both the colonial approach to data collection and methodology and the self‐inflicted pressure to make the “right observation”. From their observations, students generated hypotheses: as expected, the observations and hypotheses reflected western‐science perspectives and contained plenty of facts and measurements and absolutely no opinions or feelings or genuine desire to discover more, as that is the sole perspective that most of the students have been exposed to (Snively and Corsiglia 2001).

As part of the course objective is to consider an Indigenous paradigm in conjunction with western science approach, the instructors met with each student individually to encourage them to explore their observations and hypotheses in the context of the *Witness to the Rain* chapter and consider why they had made that particular observation, how they felt about their investigative question, and how to expand the possible outcomes and contemplate alternative explanations. Their discomfort with this different approach to the scientific method was palpable.

The “Land as Our Textbook” learning task gives the students the opportunity to learn with more than just their mind—to bring their heart along for the ride and is, by far, the assignment that the students find the most difficult. It comes in the middle of the course and the fresh vulnerability of learning how to do science in a new way makes them unsure and nervous: years of being told to omit all feeling from their scientific observations and writing is a way of learning and being that needs to be deconstructed and learned differently. In the chapter, *Mishkos Kenomagwen* (157–166), Kimmerer (2013) elegantly combines story‐telling, sensory descriptions, and the importance of listening to the teachings of the sweetgrass with experimental design. This chapter beautifully illustrates the push and pull, the braiding and unbraiding of western and Indigenous knowledge's and differing worldviews, the arrogance and assumptions of many western‐trained academics and the clear lesson to be respectful and listen to the land. This was the goal of the “Land as our Textbook” assignment.

### 2.5 Plants as Our Relatives

During the hikes mentioned above, we would stop and identify various native plants, reviewing the taxonomic information the students had learned in previous ecology courses, and also including Indigenous knowledge and information. Sometimes this was restricted to documented usage of parts of a plant, taken from western field guides and texts, such as the powder on a trembling aspen ( *Populus tremuloides* , Blackfoot otsipiiis ni) providing protection from the sun when applied to our skin, or how chewing a slice from its vascular cambium could spell off a headache (Brown 1954; Johnson et al. 2017). Other times we would share teachings gifted to us by Knowledge Holders such as the application of a poultice of Wolf Willow ( *Elaeagnus commutata* , Blackfoot misisaimi'soyiis) to an open wound to help it heal quickly (personal communication, Harley Bastien). As part of a relational learning task called Plants as our Relatives (described in detail in Cooper et al. 2025), each student selected a plant and spent time engaging with it, getting to know it better and exploring their personal connection and how they were related. The students were given the honor and responsibility of introducing the rest of us to their plant relative. We all learned to move from memorizing Latin names, genus, and species and facts to being quiet, listening, and connecting to the trees and the plants, and getting to know our plant relatives (Joseph 2021; Cooper et al. 2025). The students embraced the idea of learning from the plants rather than about them, and as one student put it:

I am not studying plants. I will not be identifying as many as I can or looking at growth patterns or populations. The plants are there to show me what I need. (Harley's Course, 2024)

### 2.6 Buffalo Rock Tipi Camp

One particularly poignant lesson that emerges in Common Ground each year is the gift of unstructured time. During the second week of the course, the students spend 3 days at Buffalo Rock Tipi Camp on the Piikani Nation next to Napii eek stunn [Oldman River] working with Knowledge Holder Harley Bastien. The students sleep in tipis set up on the grassland next to the river and spend time talking with and learning from Harley. Our days at Buffalo Rock are fluid—time is unstructured and the rising and setting of the sun are our only clock. As one student reflected:

To be completely honest, it is much different than I thought. I imagined a full itinerary, very strict and structured and full of lessons. It is much more flexible and there is more freedom to explore the camp. (Harley's Course, 2022)

Some of the students quickly settle into and appreciate this unstructured time while others really struggle. One student with anxiety found the lack of a detailed agenda excruciating. For another student, a varsity athlete whose days start early and end late with every single minute in between precisely planned and executed, the lack of structure was jarring.

Being an athlete, time management is one of my biggest strengths. Along with this comes an internal schedule. The minute I wake up I am thinking about what I need to do, what time it is, where I need to be when. My mind is always thinking about what's next. My google calendar is embedded in my brain. But [at tipi camp] I was able to relax. My only job was to be present. Which is clearly something I am not good at. I don't know what my body feels like in a state of calm. (Harley's Course, 2024)

This student's words were a stark reflection on both the general society and university students' busy lives in that the ability to ‘just be’ was so meaningful.

**FIGURE 3:**
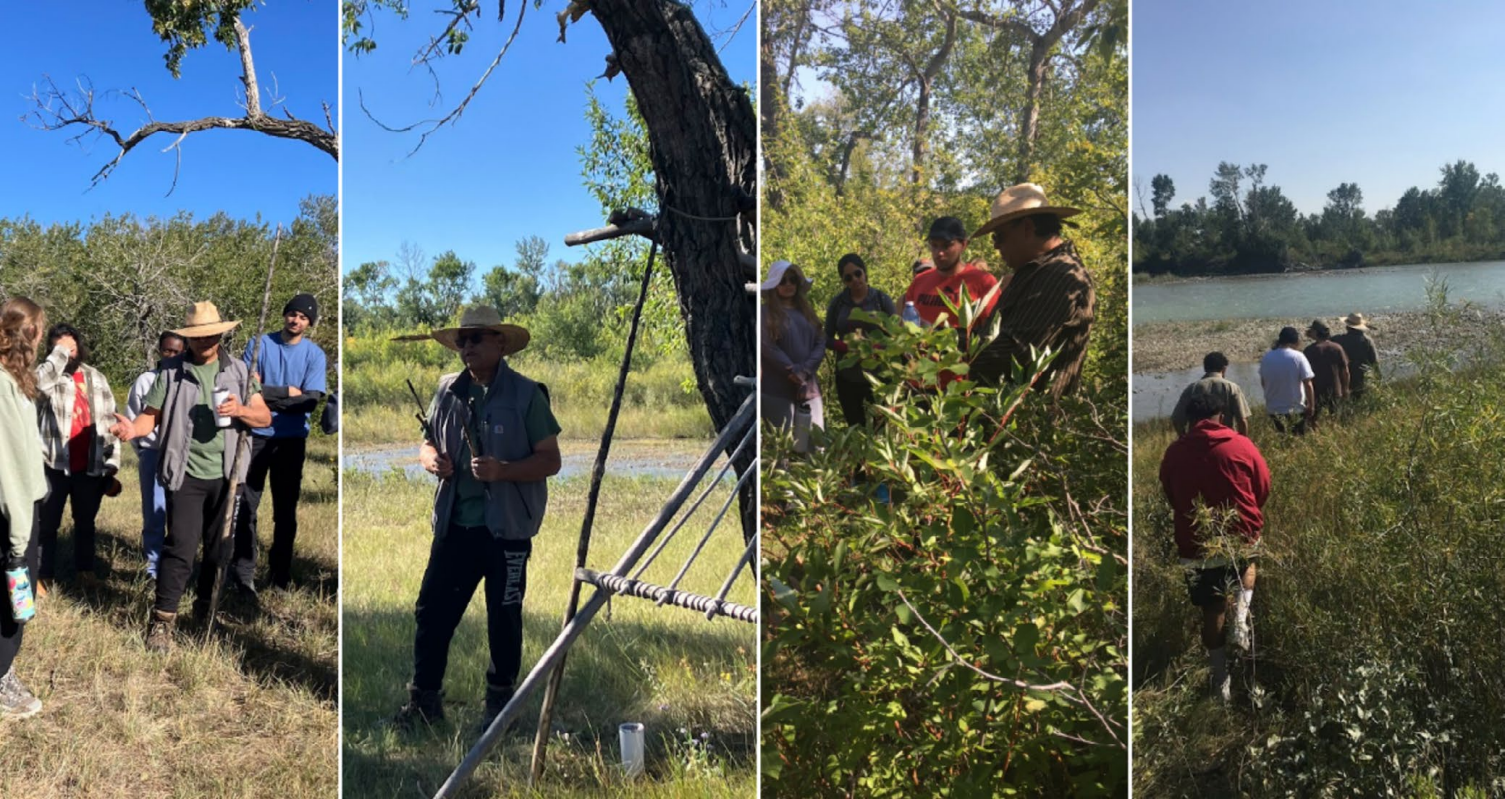
Learning from Piikani Knowledge Holder Harley Bastien at Buffalo Rock Tipi Camp.

The time spent learning from Harley at Buffalo Rock Tipi Camp is an incredibly special part of this course (Figure 3). From chats around the campfire on the importance of broadening perspective and being stewards of the land, to nature walks along the sacred shores of Napii eek stunn [Oldman River], the students' comments on this interaction speak to the powerful impact it has on them:

I loved our walk with Harley and him showing and explaining/talking about perspective—it really puts lights on certain situations. You really have to think about more than yourself: don't have tunnel vision! Having perspective can make you feel not lost or have purpose in your life is what I understood when Harley was talking. (Harley's Course, 2024)

Our walk with Harley was so amazing. The amount of knowledge and wisdom he carries is just amazing. His passion for the land and for his people is undeniable. (Harley's Course, 2024)

The highlight of the day for me was our walk with Harley. His connection with the land and his history is beautiful and I wish so much more of the world could get a chance to hear him speak. (Harley's Course, 2024)

The best thing that Harley said is that ‘nature is there for anyone who seeks it’. This point will have a special place in my life from now on. (Harley's Course, 2024)

Like Harley said, the land is him. It is a part of him. It is his family. We can't disconnect ourselves from the Earth. Our science and our Earth are what make us. (Harley's Course, 2024)

Likewise, the interaction with the students impacted Harley as well. When he read the students' comments about their time on the land at tipi camp, he said:

Thank you for sharing the students' comments with me. I am so very blessed to know that in some way Buffalo Rock has enriched some of their lives. I shed many tears reading their thoughts. May the Force of Nature be with you all. (Harley Bastien, 2024)

Being on the land, and spending time at Buffalo Rock Tipi Camp, was a critically important element of bridging Indigenous and western ways of learning science. Taking students away from the city and cell phone service, and all the demands on their time and attention, and immersing them in nature resulted in a synergistic awakening of mind and body and emotion and spirit: a holistic understanding and broadening of perspective. We are reminded of the well‐known quote by the Senegalese forestry engineer and environmentalist, Baba Dioum, who said “In the end we will conserve only what we love; we will love only what we understand; and we will understand only what we are taught” (Dioum 1968). With land‐based learning our students learn to connect with the natural world, to see the plants, and the animals, the water and the rocks, not as generic natural resources, but rather as being entities that are interconnected to each other, and to themselves. In previous ecology classes, students learned “appropriate” terminology of “biotic” and “abiotic” components of an ecosystem but they came away from this course with new found perspective to respect it, and in that a desire to nurture these relationships, and to protect these entities.

## 3 Spirituality in Science

Students who experience this course often feel uncomfortable in that this field course is different from any other course they had taken and also different from the “fun in the sun” they expected. During the 2 weeks they spend together on the land, they move from the ingrained belief and habitual approach to science that includes an unwillingness to incorporate emotions or the unfamiliar, to a state of hesitancy where they realize the limitations of science as they know it. One noticeable change, as the students are exposed to the Indigenous worldview and alternate ways of coming to know, is that they begin to acknowledge the spiritual realm, an important part of the Indigenous paradigm as shown by this student's comment:

When walking through the forest with Harley, he stopped at a point where multiple trees were bending and curving towards the earth. Harley explained that the forest floor is sacred, and the trees were reaching towards the energy of that space. In western science, the reason for the curvature of the tree could be explained by environmental conditions like heavy rain or snow, or by phototropism and maximizing solar irradiation. If the lens used to explain the bent trees was that of western science, the conclusion would not be spirituality or sacredness. But that lens would not be able to encapsulate the emotional feeling we felt while in that space, or the goosebumps on Harley's arms or how my classmates and I felt transformed after spending time there. (Harley's Course, 2024)

The students also commented on the fact that this course was the first time they ever thought about how their spirit and emotion contributed to their understanding. Spending 10 days in nature offers numerous opportunities for students to experience the interconnectedness that is present throughout an Indigenous worldview but until they took “Harley's Course”, it was only a theoretical notion rather than a lived experience. As an example, one evening at Buffalo Rock, without the light pollution from the city, the stars were dancing for us. The students lay on the ground and watched the night sky. One student wrote in her journal about this experience:

It was beautiful. [Kainai Elder] referred to the milky way as the wolf's tail and how energy can notbe created or destroyed, only transferred. I felt re‐energized from the sky. (Harley's Course, 2022)

**FIGURE 4:**
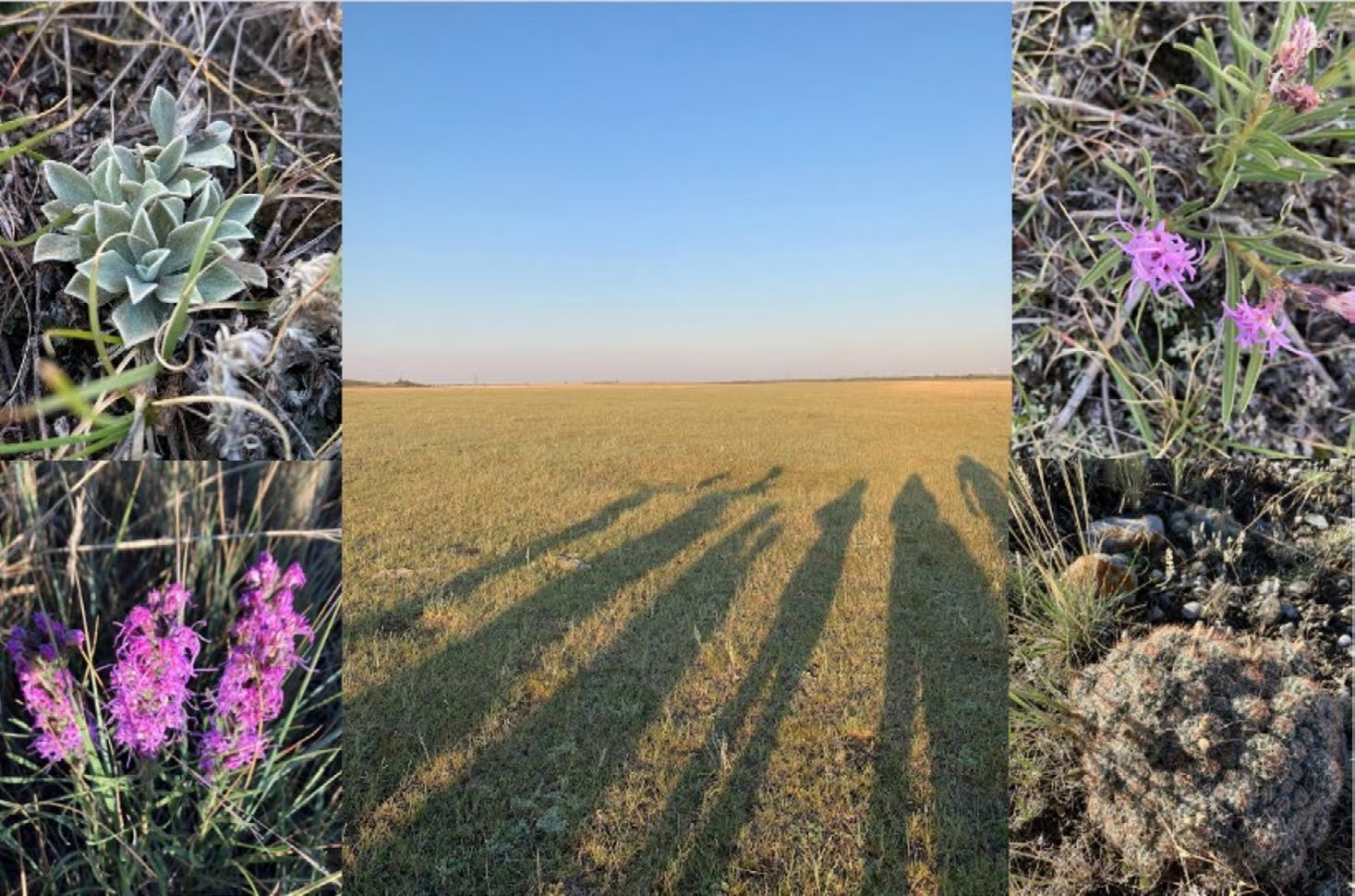
The ‘perfect’ circle formed by standing on the tipi rocks found in the native grasslands of Buffalo Rock Tipi Camp.

On an evening walk across a grassy field full of wa'piisi [Buffalo Wallows], otahkóóttsiiksi [Prickly Pear Cactus, *Opuntia* sp.], and aakííka'ksimiiyistsi [Fringed Sage, Artemisia frigida], the students stumbled across some old, half‐buried, lichen‐covered rocks. We asked each of them to find a rock and stand on it, and the surprise and wonder they expressed when they recognized the resulting perfect circle they had formed (Figure 4) was a greater experiential lesson than any words or lectures about the tipi rings that have remained undisturbed for generations. As a student said:

When we found the tipi rings and we each stood on a rock I got chills over my body. This deep realization how sacred the land we were walking on was. (Harley's Course, 2023)

One theme that came up as students reached the end of the course, was an acknowledgment that there was both discomforts, in hearing difficult stories that made them questions their ideas and in the realization that their knowledge of science is confined to mind and body, but also comfort as they took in their surroundings with a broader perspective.

### 3.1 Lessons and Challenges

The students' words and descriptions communicate the extraordinary experience of taking part in “Harley's Course”. However, it would be misleading to suggest that there have not also been challenges and difficulties over the past 3 years. With hindsight, these can be broadly grouped into four main categories: being present, being outside, being respectful, and colonization.

- *Being present*: During the first few days of the course, there is a reluctance for students to connect to the natural world, and even those that enjoy and appreciate nature through camping or hiking express that they feel disconnected or uncomfortable and view nature as a backdrop or something you observe or put in a jar. There is also uncertainty: “Harley's Course” is immersive and students do not just “take” this course, they experience it. This means a 100% commitment to the 10 days which challenges students who are used to the flexibility and concessions often given in traditionally taught mainstream courses. In order for this to happen, it seems that students have to first resign themselves to the immersive, experiential learning. In one particular instance, a student requested the flexibility to miss a few days of the course, and leave tipi camp early, in order to attend cheerleading practice. The practice was an important one and attendance was required if she was going to cheer at a high‐profile football game on the long weekend. Our response –*we would love to have you as part of the course, but we also understand if you choose to withdraw so that you can attend practice*‐ was difficult for her to receive but in the end she chose to remain in the course. On the last day of class, this student was sharing what an incredible experience it had been, and we asked how she was feeling about missing practice and not being allowed to cheer at the big game. She told us that, as it turned out, practice had actually been canceled and everything had worked out—as she said this she paused, suddenly aware of the serendipitous nature of this. She turned to us and said that she felt that she had been given a gift—one that came to her “through no action of [her] own, free, [moving] toward [her] without beckoning. It [was] not a reward; you cannot earn it, or call it to you, or even deserve it. And yet it appears. Your only role is to be open‐eyed and present” (Kimmerer 2013, 23–24), and expressed her gratitude and wonder that things had worked the way that they did.
- *Being outside*: Taking students into the elements for outdoor experience relies on their preparedness, proper clothing, and fitness levels, all of which are difficult to control. There have been wasp stings/allergies, food intolerances, wildlife (of the large, black 4‐legged carnivorous variety namely *Ursus americanus* (black bears), Blackfoot sikohkiaayo), sunburn, blisters, extreme weather, and injuries. Some students struggled on hikes and nature walks, which made for much longer travel times and negative attitudes. Despite the clear wording in the course description requiring preparedness both physically and mentally for strenuous and prolonged outdoor activities in any weather conditions, there were students that registered who were lacking in these.
- *Being respectful*: Acknowledging and accepting different perspectives takes conscious effort. We ask students to sign a contract of commitment that reminds them to keep an open mind, regardless of their own opinions or belief systems, and to adhere to the institutional code of conduct. In real time, however, this contract is just a piece of paper and does not hold much weight against a student's reaction to an assumed challenge to their religious conviction, or against dismissive offhand comments or inappropriate questions from classmates, or against the expression of engrained colonial beliefs.
- *Colonization*: As one student astutely commented, there is “much to learn and undo in the ways that [we] understand science” (Harley's Course, 2023). As non‐Indigenous instructors, we (CA and AF) sometimes feel that we have failed to teach “a science deeper than data” (Kimmerer 2013, 221). As western‐trained teachers we consider it our role to dictate the learning environment of the classroom. We set class rules and policies, provide information, assess student retention of that information, and assign grades. “Harley's Course” is about letting go—simply doing our best to guide students to use mind, body, and spirit and letting go of measurable course objectives and control—and as our mentor and colleague states, “getting it wrong at times will inevitably happen” (Hogue 2025). Perhaps the most critical lesson learned from teaching “Harley's Course” is that we are all on our own journeys.

## 4 Conclusion

Coordinating, organizing and running “Harley's Course” takes a great deal of energy, time, and intention. As part of the academy, it often feels that “getting scientists to consider the validity of Indigenous knowledge is like swimming upstream in cold, cold water” (Kimmerer 2013, 160). The implementation of the C^4^‐R^4^ methodological approach of Co‐learning Co‐designing Co‐creating and Co‐sharing as Indigenous and non‐Indigenous scientists while maintaining Respectful relationships, cultural Relevance, Responsibility and Reciprocity (Hogue 2025) is essential to navigating this space and doing this work in a good way. We also lean on the support offered by Mi'kmaw Elders Albert and the late Murdena Marshall, and biologist Dr. Cheryl Barlett whose encouragement to “weave indigenous ways of knowing and knowledge systems into today's post‐secondary educational curricula for sciences” (Bartlett et al. 2012, 340) through Two‐Eyed Seeing is a foundational motivator of this course. The most important conclusion is that the student impact from this course is immeasurable. Each year, we are humbled to read comments from the students that speak to the powerful experience of immersing themselves in this course:

I feel like in this two week period I've learned more than I have in full semester of regular classes. (Harley's Course, 2022)

I feel like I am bursting with questions. I feel like my eyes are finally open. (Harley's Course, 2022)

This course has flipped my perception of my identity on its head. (Harley's Course, 2022)

Maybe this was a class I was meant to take, a view I was destined to consider and that is the real reason I have the privilege of being in this course. (Harley's Course, 2023)

To think of my past is to go against how I have tried to live my life thus far. I like to spend most of my time keeping myself busy. I tell myself that I do this to occupy myself but deep down I realize it is to keep me from thinking too hard about my past and where I come from. I always knew a time would come where the long‐hour work weeks would no longer shield me from thinking of my life to this point – I just didn't expect it to be in a 3rd year block week course. (Harley's Course, 2023)

One of the stories the students read in their textbook *Braiding Sweetgrass: Indigenous Wisdom, Scientific Knowledge and the Teaching of Plant* (Kimmerer 2013) is about lichen mutualisms, and how putting the algae and the fungi in the petri dish together did not ensure they would “meld” into the glorious mutualistic relationship that they exist in. Recently, a third member to the mutualism has been described ‐ a single‐celled yeast that likely provides the CO_2_ to the algae (Spribille et al. 2016, 488). Metaphorically, it feels as though at the beginning of “Harley's Course’, the instructors are the fungi, attempting to wrap our mycelial arms around the students' minds and permeate their spirits, to protect them just enough to allow them the self‐confidence to get their algae‐selves committed to engaging in the course. The elusive single‐celled yeast is their own hidden sense of their spirit. This tri‐mutualism is necessary because then and only then, could the students' energy contribute to and feed the development of the course, and feed their personal development which is necessary to be able to see beyond the conventional science courses they are accustomed to and allow themselves to truly engage with and experience the experiential learning opportunity.

In summary, our experience teaching “Harley's Course” over the past 3 years has been that we can—and should‐ plant the seeds to broaden perspective and consider the intersection between Indigenous and western ways of knowing. For some, the seed coat is thick and it takes longer to germinate. For some, the seed may not germinate (maybe the soil lacks the “resources” to nourish it (closed minded)) or it may take ten more seeds to increase the probability that at least 1 or 2 will be successful. Perhaps germination success is affected by each individual's own life experiences. In nature, many seeds require freezing, or scarification, to make the seed coat more permeable. For example, having evolved with frequent forest fire events, the serotinous cones of the Lodgepole Pine ( *Pinus contorta* , Blackfoot apahtó ‘kii’) will only germinate upon exposure to extreme heat. Regardless, for many, the opportunity to participate in a field course such as this has immeasurable and long‐lasting impact. We are grateful to Harley Bastien for his leadership and teachings and for upholding the spiritual integrity of the land. We are grateful for all of the students that took a chance on “Harley's Course”, for the support of the Faculty of Science and Technology at Mount Royal University, for the Blackfoot Elders and Knowledge Holders that have shared their teachings and for the magnificent and storied land of Treaty 7 Territory.

## Author Contributions

**Carol L. Armstrong:** conceptualization (equal), methodology (equal), visualization (equal), writing – original draft (lead), writing – review and editing (lead). **Alexandria Farmer:** conceptualization (equal), methodology (equal), writing – original draft (equal), writing – review and editing (equal). **Michelle Hogue:** conceptualization (equal), writing – review and editing (equal). **Harley Bastien:** conceptualization (equal), writing – review and editing (supporting).

## Conflicts of Interest

The authors declare no conflicts of interest.

## Data Availability

The authors have nothing to report.
